# Pumpkin Seed Oil-Loaded Niosomes for Topical Application: 5α-Reductase Inhibitory, Anti-Inflammatory, and In Vivo Anti-Hair Loss Effects

**DOI:** 10.3390/ph15080930

**Published:** 2022-07-27

**Authors:** Veerawat Teeranachaideekul, Warisara Parichatikanond, Varaporn Buraphacheep Junyaprasert, Boontida Morakul

**Affiliations:** 1 Department of Pharmacy, Faculty of Pharmacy, Mahidol University, Bangkok 10400, Thailand; veerawat.tee@mahidol.edu (V.T.); varaporn.jun@mahidol.ac.th (V.B.J.); 2 Department of Pharmacology, Faculty of Pharmacy, Mahidol University, Bangkok 10400, Thailand; warisara.par@mahidol.edu; 3 Center of Biopharmaceutical Science of Healthy Ageing (BSHA), Faculty of Pharmacy, Mahidol University, Bangkok 10400, Thailand

**Keywords:** pumpkin seed oil, niosomes, anti-hair loss, 5α-reductase

## Abstract

Pumpkin seed oil (PSO)-loaded niosomes were prepared from Tween 20 and cholesterol by ethanol injection. Confocal microscopy showed better skin permeation and hair follicle accumulation of the niosomes compared to the PSO solution. The PSO-loaded niosomes inhibited 5α-reductase activity in DU-145 cells and hindered IL-6 activity in RAW 264.7 cells. These effects indicated the great potential of PSO-loaded niosomes to reduce hair loss. The hair scalp serum with PSO-loaded niosomes did not show irritation to reconstructed human skin. This formulation presented a significant decrease in the percentage of fallen hairs by 44.42% in the in vivo 60-second hair count experiment and a significant increase in the anagen to telogen (A/T) ratio (1.4-fold) in the TrichoScan^®^ evaluation after 8 weeks of treatment compared to the initial conditions, indicating the promising efficacy of PSO-loaded niosomes as a natural alternative for anti-hair loss therapy.

## 1. Introduction

Hair loss and androgenetic alopecia cause direct distress regarding self-confidence, affecting the individual’s quality of life [1]. Androgenetic alopecia is characterized by follicular miniaturization in a patterned hair loss, occurring due to systemic androgen and genetic factors [2,3,4].

Androgen regulates the change of vellus hairs to terminal hairs which are longer, thicker, and darken in color [5]. Androgenetic alopecia is noticed as a slow transformation of terminal hair follicles to shorter, thinner, and shallower vellus hair with a much shorter anagen phase [6]. Testosterone is a major male androgen associated with hair loss. Testosterone can be converted to androgen dihydrotestosterone (DHT) which is more potent than testosterone by the enzyme 5α-reductase in combination with NADPH as a cofactor prior to binding with the androgenic receptor [7]. Furthermore, transcription of androgen-dependent genes produces baldness [8,9]. Therefore, one effective mechanism to treat hair loss is to inhibit the activity of the enzyme 5α-reductase, leading to an anti-androgen effect and subsequent reduction in hair loss.

Finasteride is the first-line drug used to treat hair loss that functions as a 5α-reductase type II inhibitor [10]. However, some drawbacks of using finasteride have been reported, such as sexual effects including erectile dysfunction, ejaculatory dysfunction, and loss of libido [11,12,13,14]. The undesired side effects encourage individuals suffering from hair loss to seek alternative and efficient treatments with fewer side effects, especially plant and herbal treatments. Pumpkin (*Cucurbita pepo* L.) is a plant whose seeds exhibit various medicinal properties. The composition and contents of fatty acids, tocopherols, and sterols in pumpkin seed oil (PSO) provide positive health effects [15,16,17], including against hypertension [18], carcinogenic diseases [19], antidiabetic [20], antibacterial [21], antioxidant [16], anti-inflammatory, and wound healing properties [22]. Moreover, it can be used for benign prostatic hyperplasia [23] and to help relieve bladder and prostate discomfort and reduce inflammation in the prostate due to its diuretic effect. A previous study reported that the major active compound found in PSO that is responsible for its effects to treat benign prostatic hyperplasia is β-sitosterol [24]. It has been reported that β-sitosterol inhibits the growth and migration of prostate cancer cells and tumors in mice, which was suggested to occur by an androgenic mechanism. β-sitosterol acts as an inhibitor of 5α-reductase, the enzyme that converts testosterone to DHT [25,26]. Therefore, β-sitosterol, which is an active component in PSO, might also be of interest for use as an alternative treatment for androgenetic alopecia. 

The preparation of topical formulations for the treatment of hair loss should provide good permeability through the skin and high accumulation in hair follicles. In previous studies, microparticles and nanoparticles have been reported to provide higher accumulation in hair follicles than solutions after application to the hair scalp. Therefore, the encapsulation of active compounds into nanoparticles or microparticles could improve hair follicle targeting [27,28]. Nanoparticles have been used as drug delivery systems to improve skin penetration [29]. One type of nanoparticles of interest for skin delivery is niosomes. When compared with liposomes, niosomes show the better chemical stability, no requirement of special condition in preparation and storage, and low production cost. Niosomes have a lamellar structure and microscopic size when used for drug delivery. They usually consist of nonionic surfactants and cholesterol and form bilayer vesicles that can incorporate both hydrophilic and hydrophobic active components [30,31,32]. In the present study, the PSO-loaded niosomes were developed from our previous study [33]. By varying the types and amounts of various components, the PSO-loaded niosomes prepared from PSO, Tween 20, and cholesterol at the ratio of 2:2:1 by weight showed the smallest particle size with narrow size distribution and good stability. Therefore, this formulation was selected for further evaluation. In the present study, we aimed to investigate whether the incorporation of PSO into niosomes might help to increase the permeability of PSO through the skin and improve PSO accumulation in hair follicles. A PSO-loaded niosome formulation was prepared to provide increased skin permeation and hair follicle accumulation, which were investigated using confocal microscopy on pig ear skin. The effect of PSO-loaded niosomes against anti-hair loss by inhibiting 5α-reductase and reducing inflammation related to the inhibition of interleukin-6 (IL-6) was evaluated. Moreover, hair scalp serum containing PSO-loaded niosomes was developed. The in vivo efficacy of the hair scalp serum containing PSO-loaded niosomes to reduce hair loss was evaluated in panelists via a 60-second hair count test and a phototrichogram using TrichoScan^®^ [34,35]. The skin irritation caused by the product was also examined using a reconstructed human skin model. This study aims to develop and evaluate the anti-hair loss efficacy of PSO-loaded niosomes to consequently provide evidence for further evaluation as an alternative hair loss treatment. 

## 2. Results and Discussions

### 2.1. Skin Permeation and Hair Follicle Accumulation

**Figure 1:**
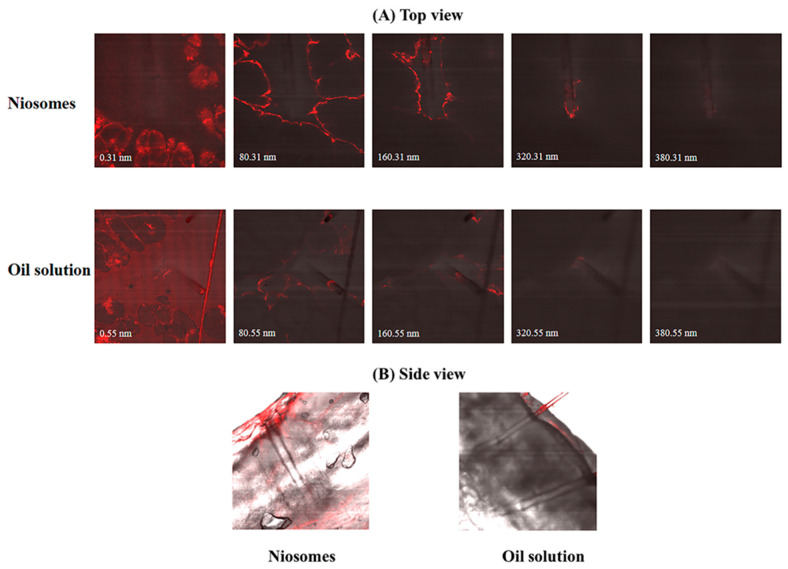
Confocal microscopy images indicating the penetration of Nile red-loaded niosomes across the excised pig ear skins in top view (**A**) and side view (**B**) compared with the control (Nile red oil solution).

Figure 1 shows confocal images of the pig ear skins after applying Nile red-loaded niosomes and Nile red oil solution in both the top view (A) and side view (B). It was found that Nile red was detected in the deeper skin and accumulated in the hair follicles of excised pig ear skins after applying the Nile red-loaded niosomes. In the top view, Nile red was detected until at a depth of approximately 320 nm and approximately 160 nm after application of niosomes and oil solution, respectively. Given the fact that niosomes have lamellar structures of nonionic surfactants and cholesterols, they could help improving the permeation of active components through the skin. Moreover, previous studies have reported that microparticles and nanoparticles can accumulate in hair follicles after being applied to the hair scalp. As a result, both microparticles and nanoparticles can be used as a delivery system for hair follicle targeting [36,37,38]. From the results, it might be inferred that the oil-soluble actives in niosomes had an improved possibility to permeate and accumulate in the hair follicles compared to the normal oil solution. 

### 2.2. Evaluation of the Anti-5α-Reductase Activity in DU-145 Cells

#### 2.2.1. Cell Viability

**Figure 2:**
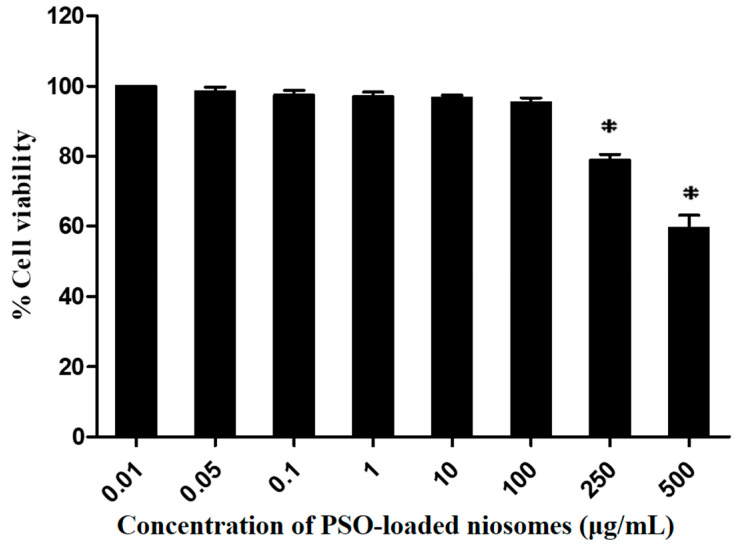
Percent viability of DU-145 cells after incubation with PSO-loaded niosomes in the concentration range of 0.01–500 μg/mL for 24 h. * Significant reduction in percent cell viability (*p* < 0.05).

The cellular viability of PSO-loaded niosomes was evaluated using the 3-(4,5-dimethylthiazol-2-yl)-2,5-diphenyl-2H-tetrazolium bromide (MTT) assay in DU-145 cells. The concentration of the sample was varied from 0.01–500 μg/mL to select an appropriate concentration for further use in the 5α-reductase inhibition study, which is associated with the anti-hair loss properties. The DU-145 cell viability after incubation with PSO-loaded niosomes in the concentration range of 0.01–100 μg/mL for 24 h remained higher than 80% and no significant difference among concentrations was observed (*p* > 0.05). Moreover, incubation of DU-145 cells with PSO-loaded niosomes at concentrations of 250 and 500 μg/mL showed significant decreases in cell viability to 79.8 ± 3.3% and 59.5 ± 7.3%, respectively (*p* < 0.05) (Figure 2). Therefore, 100 μg/mL PSO-loaded niosomes was used for further study (the anti-5α-reductase activity) because it was the highest concentration that did not cause cytotoxicity.

#### 2.2.2. Anti-5α-Reductase Activity

**Figure 3:**
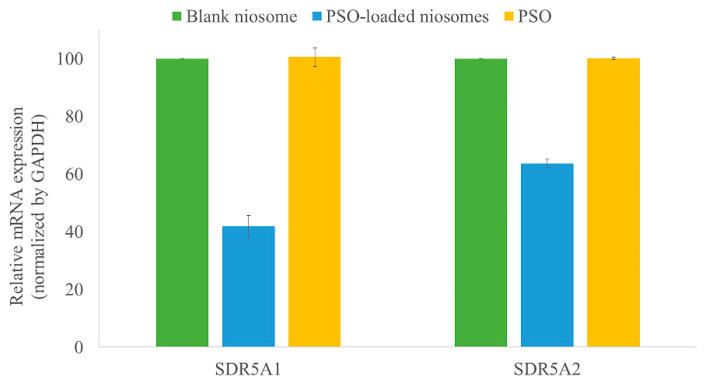
Relative mRNA expression of the SRD5A1 (upper) and SRD5A2 (lower) genes in DU-145 cells after incubation with PSO-loaded niosomes, blank niosomes, and pure PSO. PSO-loaded niosomes inhibited the mRNA expression of the SRD5A1 and SDR5A2 genes compared to the blank niosomes and pure PSO.

Anti-5α-reductase activity was evaluated by measuring the mRNA expression of hair loss genes (SRD5A1 and SRD5A2) in DU-145 cells by real-time polymerase chain reaction (qPCR). The ability of 100 μg/mL PSO-loaded niosomes to restrain the expression of the SRD5A1 and SRD5A2 genes was evaluated and compared with those from the blank niosomes and pure PSO at a similar concentration, as shown in Figure 3. The results indicated that PSO-loaded niosomes inhibited the mRNA expression of the SRD5A1 and SRD5A2 genes by 41.84 ± 3.69% and 63.57 ± 1.48%, respectively, compared to the blank niosomes. Pure PSO showed no inhibitory activity on the mRNA expression of the SRD5A1 and SRD5A2 genes. During the experiment, it was found that the addition of pure PSO to DU-145 cells resulted in oil separation and the formation of a thin, oily film on the surface of the cells. As a result, pure PSO could not permeate into the cells. Therefore, pure PSO was not able to inhibit the mRNA expression of the SRD5A1 and SRD5A2 genes. From the obtained results, it could be clearly explained that the PSO-loaded niosomes could improve the permeability of PSO through the cells and inhibit mRNA expression of the SRD5A1 and SRD5A2 genes. As known, SRD5A1 and SRD5A2 involve in the synthesis of the enzyme 5α-reductase; therefore, hair loss reduction might be achieved with the use of PSO-loaded niosomes.

### 2.3. Evaluation of Interleukin-6 Inhibition Activity

#### 2.3.1. Cell Viability

**Figure 4:**
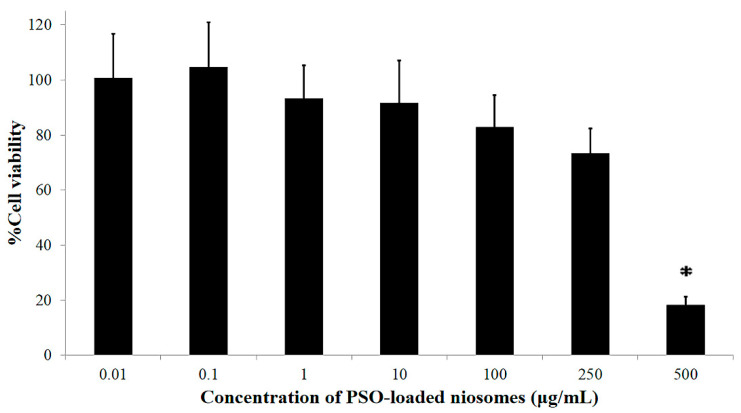
Percent viability of RAW 264.7 cells after incubation with PSO-loaded niosomes in the concentration range of 0.01–500 μg/mL for 24 h. * Significant reduction in percent cell viability (*p* < 0.05).

The cellular viability of PSO-loaded niosomes was evaluated using the MTT assay in RAW 264.7 cells. The concentration of the sample was varied from 0.1–500 μg/mL to select an appropriate concentration for further use in the interleukin-6 inhibition study, which is associated with the anti-inflammatory effect. The cellular viability of RAW 264.7 cells after incubation with 0.01–100 μg/mL PSO-loaded niosomes for 24 h remained greater than 80%. However, an increase in the concentration of PSO-loaded niosomes higher than 100 μg/mL led to a reduction in cell viability. It was found that cell viabilities of 73.4 ± 9.1% and 18.2 ± 3.0% were observed when RAW 264.7 cells were exposed to 250 and 500 μg/mL PSO-loaded niosomes, respectively, as shown in Figure 4. Therefore, the concentration of 100 μg/mL of PSO-loaded niosomes was used for the interleukin-6 inhibition study.

#### 2.3.2. Anti-Inflammatory Assay (IL-6 Inhibition)

Hair loss can be caused by inflammation of the scalp skin and hair follicles [39]. Hence, the anti-inflammatory effect was studied to observe the ability of the active components to treat hair loss. Based on Table 1, pure PSO solution in 10%v/v DMSO and the PSO-loaded niosomes at a concentration of 100 µg/mL were able to inhibit IL-6 production in LPS-induced RAW 264.7 cells by 99.55 ± 0.02% and 94.19 ± 0.86%, respectively, compared with the positive control (LPS-induced RAW 264.7 cells without treatment). The IL-6 inhibitory activities of PSO in DMSO solution and niosomes were higher than that of the standard anti-inflammatory agent (1000 µM diclofenac sodium solution). From the obtained results, the PSO solution and PSO-loaded niosomes presented an anti-inflammatory effect that might be useful for combating localized inflammation around hair follicles. Moreover, it might be assumed that the PSO solution and PSO-loaded niosomes could be used to treat hair loss caused by scalp skin and hair follicle inflammation.

### 2.4. Preparation and Characterization of the Hair Scalp Serum Containing PSO-Loaded Niosomes

The optimized formulation of hair scalp serum containing PSO-loaded niosomes was characterized based on its appearance with an organoleptic test. The formulation had a light texture, translucent white color, and aromatic odor. No separation or precipitation was observed. The pH of hair scalp serum containing PSO-loaded niosomes was 5.5 ± 0.1 and the viscosity was 39.1 ± 0.6 cps. The formulation showed good stability without changes in appearance, pH, or viscosity during 3 months of storage at 40 °C. The stability of niosomes after reconstitution into the hair serum was also investigated by measuring the particle size of niosomes using Zetasizer Nano ZS. After 3 months of storage, it was found that the size of niosomes in the hair serum slightly increased from 195.13 ± 1.41 nm to 256.27 ± 3.98 nm. The increased size of niosomes might be due to the particle aggregation or association with other components in the serum.

### 2.5. Evaluation of Skin Irritation in Reconstructed Human Skin Model

**Figure 5:**
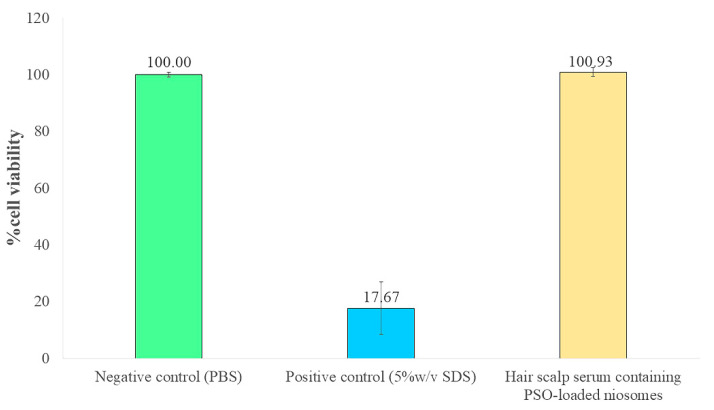
Percent cell viability in the reconstructed human skin model (EpiSkin^TM^) after incubation with hair scalp serum containing PSO-loaded niosomes for 24 h compared with the negative control (PBS) and positive control (5% *w*/*v* SDS).

Skin irritation caused by the hair scalp serum containing PSO-loaded niosomes was evaluated using a reconstructed human skin model. After incubation of the hair scalp serum containing PSO-loaded niosomes on the reconstructed human skin for 24 h, the cell viability was 100.93 ± 1.50%. As a result, the hair scalp serum containing PSO-loaded niosomes was classified as a nonirritant to skin because they provided higher than 50% cell viability [40]. Therefore, this formulation is suitable for application on the skin (Figure 5).

### 2.6. In Vivo Study of the Anti-Hair Loss Activity of Hair Scalp Serum Containing PSO-Loaded Niosomes

The 60-second hair count study was performed on 42 panelists before and after using the test product for 4 and 8 weeks. The results showed that after using hair scalp serum containing PSO-loaded niosomes, the reduction in the number of fallen hairs was significantly lowered to 23.57 ± 1.29% and 44.42 ± 1.22% after 4 and 8 weeks, respectively (*p* < 0.05), as shown in Table 2. Additionally, 64.29% and 80.95% of the panelists were found to have a reduction in the number of fallen hair after 4 and 8 weeks, respectively. 

**Figure 6:**
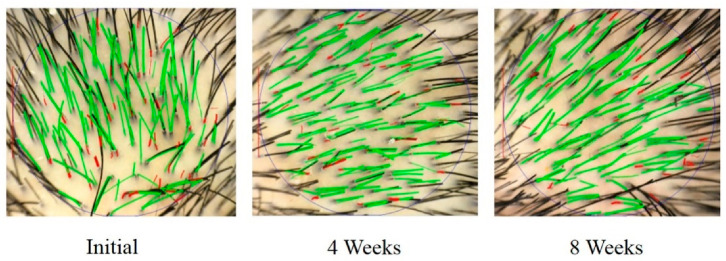
TrichoScan^®^ photographs of the panelist at the initial time point and after using the hair scalp serum containing PSO-loaded niosomes for 4 and 8 weeks.

The TrichoScan^®^ evaluation was performed with 8 panelists before and after using hair scalp serum containing PSO-loaded niosomes for 4 and 8 weeks. Anagen is the active growth phase of hair follicles. During the anagen phase, the hair follicles push out hairs and continue to grow until they reach the end of their lifespan and fall out. However, the telogen phase is the resting state of hair follicles. During the telogen phase, the hair follicles are completely at rest and have stopped growing. Telogen hairs are predominant findings in alopecia. An increased A/T ratio could indicate an improvement in hair growth. The A/T ratio in each panelist was calculated before and after application of the product, and the results were compared. After using the hair scalp serum containing PSO-loaded niosomes, the A/T ratio increased. In particular, at 8 weeks, the A/T ratio was significantly increased by 1.4-fold compared to the initial conditions (without the use of hair scalp serum containing PSO-loaded niosomes) (*p* < 0.05) as shown in Table 3. An example of a phototrichogram by TrichoScan^®^ at baseline (initial) and after using the product for 4 and 8 weeks is shown in Figure 6. From the results, the in vivo 60-second hair count and TrichoScan^®^ evaluation assays indicate an anti-hair loss effect of the hair scalp serum containing PSO-loaded niosomes. Therefore, this preparation might be potential for further development as a hair loss treatment.

## 3. Materials and Methods

### 3.1. Materials

PSO was purchased from Jilin Baili Biotechnology (Changchun, China). Tween 20 was achieved from Ajax Finechem (Sydney, NSW, Australia). Cholesterol was obtained from Loba Chemi (Mumbai, India). Sterile water for injection was obtained from General Hospital Product Public Company Ltd. (Pathum Thani, Thailand). All other chemicals and reagents were of analytical grade. The human prostate cancer cell line DU-145 and RAW 264.7 cells were purchased from the American Type Culture Collection (ATCC) (Manassas, VA, USA). Dulbecco’s modified Eagle’s medium (DMEM), fetal bovine serum (FBS), nonessential amino acids (NEAAs), penicillin-streptomycin (P/S), and Hanks’ balanced salt solution (HBSS) were supplied by Life Technologies (Paisley, UK). RNA extraction for the anti-5α-reductase inhibition assay was conducted using a GeneJET RNA Purification Kit purchased from Thermo Fisher Scientific (Waltham, MA, USA). The expression levels of 5α-reductase genes were evaluated using KAPA SYBR FAST One-step RT-qPCR kits purchased from Merck KGaA (Darmstadt, Germany). The anti-inflammatory assay, which assessed the inhibition of interleukin-6 (IL-6) concentration, was conducted using a LEGEND MAXTM Mouse IL-6 ELISA Kit with precoated plates (BioLegend, San Diego, CA, USA).

### 3.2. Preparation of Pumpkin Seed Oil (PSO)-Loaded Niosomes

The development and preparation of PSO-loaded niosomes (F1) were described in our previous study [33]. Briefly, PSO, Tween 20, and cholesterol were mixed at a weight ratio of 2:2:1. The mixture was dissolved in ethanol under sonication and warmed at 50 °C for 5 min. The mixture was subsequently injected into prewarmed sterile water at 75 °C under stirring at 300 rpm. Then, a milky white dispersion was obtained. The ethanol was removed from the formulation by evaporation at 75 °C with continuous stirring at 300 rpm for 20 min to obtain PSO-loaded niosomes. The percentage of PSO loaded into the niosomes was 0.5. The PSO-loaded niosomes showed an average particle size of 172.5 nm with a polydispersity index of 0.122 and zeta potential of -33.3 mV.

### 3.3. Skin Permeation and Hair Follicle Accumulation Study

This study was conducted according to animal care and use protocol number PYR001/2019 (Institute Animal Care and Use Committee, Faculty of Pharmacy, Mahidol University). Pig ear skin was used as a model tissue because of its similarity to human skin. Pig ears were derived from pigs (Sus domesticus) that were provided by a local abattoir in Nakhon Pathom Province. The obtained pig ear skin was cleaned, and the hairs were shortened with scissors. The excised pig ear skins were mounted on Franz diffusion cells to control the temperature at 32 °C to mimic the human skin surface temperature. The excised pig ear skins were equilibrated for 30 min before application of the test samples. After equilibration, 30 μL of the test samples containing the oil-soluble fluorescent dye (Nile red) were applied simultaneously to the dorsal side of the pig ear skin and massaged manually using gloved fingertips for 3 min. The excised pig ear skin was incubated for 1 h under nonocclusive conditions. The test sample (Nile red-loaded niosomes) was compared with the control (Nile red oil solution) to evaluate the efficiency of the permeation through the skin and accumulation in the hair follicles. Excised pig ear skin treated with water was used as a reference. The experiment was performed in triplicate. 

After 1 h of incubation, the pig ear skins were observed under a confocal microscope from the top and side. For the top view, the excised pig ear skin was mounted on a slide and sealed with a coverslip. In case of the side view, the excised pig ear skin was cross sectioned at a thickness between 10–20 µm vertically from the acceptor side to the donor side. Imaging was performed using an inverted confocal laser scanning microscope (Olympus FV1000, Tokyo, Japan). A 12 V laser line at 543 nm was used as the excitation source. The emitted fluorescence was detected with an Alexa Fluor filter at 546 nm. The fluorescence intensity and depth of the Nile red-loaded niosomes that penetrated through the skin or accumulated in the hair follicles were evaluated and compared with those of the Nile red oil solution.

### 3.4. Evaluation of Anti-5α-Reductase Activity in DU-145 Cells

The 5α-reductase enzyme is involved in conversion of the hormone testosterone into hormone DHT. A large amount of DHT is associated with weakness in the hair roots, a reduction in hair growth, and hair loss problems. Therefore, investigating the active components that can inhibit 5α-reductase enzymatic activity is a target method for solving the problem of hair loss. The ability of PSO-loaded niosomes to inhibit 5α-reductase activity was evaluated using DU-145 cells and the results were compared with blank niosomes and pure PSO. Several studies indicated that the DU-145 human androgen-insensitive prostate adenocarcinoma cells express the SRD5A genes associated with 5α-reductase activity [41,42]. Therefore, those cells were employed in the present study as a representative model to investigate the regulation of SRD5A gene expression against the 5α-reductase activity. Herein, the maximum concentration of administered PSO-loaded niosomes that retained greater than 80% DU-145 cell viability was evaluated and further used to study the mRNA expression of hair loss genes.

#### 3.4.1. Cell Culture

DU-145 cells were cultured and maintained in MEM supplemented with 10% FBS and 1% P/S and incubated in a humidified atmosphere of 5% CO_2_ at 37 °C. After the DU-145 cells were grown to 80% confluence, they were passaged by treatment with 0.25% trypsin-EDTA solution to maintain the exponential growth stage. The medium was changed to serum-free MEM and incubated for at least 30 min before further experiments.

#### 3.4.2. Cell Cytotoxicity Test

Cell cytotoxicity was determined using a modified MTT assay. Briefly, DU-145 cells were seeded in 96-well plates at a density of 1 × 10^4^ cells/well in 200 µL of MEM supplemented with 1% FBS and 1% P/S and incubated overnight. The cells were divided into control and treatment groups. The control group was exposed to the vehicle (water) while the treatment groups were treated with various concentrations of PSO-loaded niosomes (0.01–500 µg/mL diluted with water) for 24 h. After incubation, the culture medium was replaced with 100 µL of MTT solution and the DU-145 cells were incubated for an additional 4 h at 37 °C. Then, the MTT solution was removed by aspiration, and 100 µL of DMSO was added to dissolve the insoluble formazan product. The samples were mixed thoroughly and the optical density (OD) of each well was measured at 570 nm with a microplate reader (Infinited^®^ M200, Tecan, Männedorf, Switzerland). The results were calculated as a percentage of the treated cells compared with control cells, as shown in Equation (1). All experiments were performed in triplicate.

(1) $$\%\mathrm{Cell}\;\mathrm{viability}=\frac{\mathrm{Absorbance}\;\mathrm{of}\;\mathrm{treated}\;\mathrm{cells}}{\mathrm{Absorbance}\;\mathrm{of}\;\mathrm{control}\;\mathrm{cells}}\times 100\;\;$$

#### 3.4.3. Measurement of the mRNA Expression of Hair Loss Genes by Real-Time Polymerase Chain Reaction (qPCR)

DU-145 cell pellets were plated onto 6-well plates separately at a density of 2.5 × 10^4^ cells/well and incubated with 10% FBS and 1% P/S in a 5% CO_2_ incubator at 37 °C. Cells were then exposed to the test samples (PSO-loaded niosomes, blank niosomes, and pure PSO) at a concentration of 100 µg/mL, which was the maximum concentration that gave greater than 80% viability for 24 h. The medium was removed, and the cells were washed with PBS, trypsinized with 0.25% trypsin solution for 2 min, and suspended in PBS. 

Total RNA from DU-145 cells was extracted with the GeneJET RNA Purification Kit (Thermo Fisher Scientific, Vilnius, Lithuania) following the instructions of the manufacturer. The expression levels of 5α-reductase genes, including steroid 5α-reductase 1 (SRD5A1) and steroid 5α-reductase 2 (SRD5A2), were determined by real-time polymerase chain reaction (qPCR) using KAPA SYBR FAST One-step RT-qPCR kits according to the manufacturer’s protocol. The gene-specific primers for RT-qPCR were designed as shown in Table 4. RT-qPCR was analyzed under the following conditions: reverse transcription at 42 °C for 5 min; reverse transcriptase (RT) inactivation and DNA polymerase activation at 95 °C for 2–5 min; combined annealing, extension, and data acquisition at 95 °C for 3 s and 55 °C for 30 s (40 cycles); and the last extension step at 72 °C for 1 min followed by at 25 °C for 2 min. Relative mRNA expression levels were evaluated by the comparative cycle threshold (CT) method, normalized to the endogenous reference, glyceraldehyde-3-phosphate dehydrogenase (GAPDH), and expressed as a fold over the control group. The fold increase in mRNA expression of the target gene was calculated by 2^−ΔΔCT^. The percentages of SRD5A1 and SRD5A2 inhibition were calculated by Equation (2). The experiments were performed in triplicate.

(2) $$\%\mathrm{Inhibition}\;=\frac{\left(\mathrm{Control}-\mathrm{Sample}\right)}{\mathrm{Control}}\times 100\;$$

### 3.5. Evaluation of Interleukin-6 (IL-6) Inhibition

Localized inflammation around hair follicles is associated with hair loss [43]. Therefore, the efficiency of anti-inflammatory active components might influence the hair loss treatment. The murine macrophage cell line RAW 264.7 is an in vitro model for evaluating anti-inflammatory activity. The anti-inflammatory activity of PSO-loaded niosomes was analyzed by determining the in vitro production of the inflammatory mediator cytokine IL-6 in RAW 264.7 cells. In this study, the maximum concentration of PSO-loaded niosomes that provided a percent RAW 264.7 cell viability greater than 80% was evaluated and further used to study the anti-inflammatory activity.

#### 3.5.1. Cell Culture

RAW 264.7 macrophages (ATCC^®^ TIB-71TM) were grown in DMEM supplemented with 10% FBS, 1% NEAAs, and 1% P/S, and then incubated at 37 °C under 5% CO_2_ until the cells were 80% confluent. The cells were washed and harvested using a cell scraper.

#### 3.5.2. Cell Viability Test

RAW 264.7 cell viability was tested using a modified MTT assay. Briefly, RAW 264.7 cells were seeded in 96-well plates at a density of 1 × 10^4^ cells/well in 180 µL of cell culture medium for overnight incubation at 37 °C under 5% CO_2_. The cells were divided into control and treatment groups. The control group was exposed to the vehicle (medium), while the treatment groups were treated with various concentrations of PSO-loaded niosomes (0.01–500 µg/mL diluted with water) for 24 h. After incubation, the culture medium was replaced with 100 µL of MTT solution and the RAW 264.7 cells were incubated for an additional 30 min at 37 °C. Then, the MTT solution was removed by aspiration, and 180 µL of DMSO was added to dissolve the insoluble formazan product followed by incubation for 30 min. The samples were mixed thoroughly, and the optical density (OD) of each well was measured at 570 nm with a microplate reader. The results were calculated as a percentage of the value of the control cells, as shown in Equation (1). All experiments were performed in triplicate.

#### 3.5.3. Pro-Inflammatory Activation of RAW 264.7 Cells

The activation of inflammatory conditions in RAW 264.7 cells was conducted according to the studies by Zhu et al. [44] and Sandhiutami et al. [45]. Cells were cultured in a 6-well plate at a concentration of 1 × 10^5^ cells/well and incubated in cell culture medium at 37 °C under 5% CO_2_ for 24 h. After incubation, the culture medium was discarded and replaced with the test samples (PSO-loaded niosomes and pure PSO solution in 10%v/v DMSO) at a concentration of 100 µg/mL (the maximum concentration that gave greater than 80% cell viability). After 2 h of incubation with the test samples, 200 µL of 1 µg/mL lipopolysaccharide (LPS) from *Escherichia coli* was added to each well for 24 h of incubation at 37°C under 5% CO_2_. The medium was then taken for the IL-6 assay. Before the assay, the samples were centrifuged at 2000 rpm for 10 min. The supernatants were collected and stored at −80 °C to quantify the IL-6 concentration. A 1000 µM diclofenac sodium solution was used as the standard anti-inflammatory agent. The positive control was LPS-induced RAW 264.7 cells without treatment, and the negative control was normal RAW 264.7 cells without LPS induction. The experiments were performed with five replicates.

#### 3.5.4. IL-6 Assay

The IL-6 concentration was quantified using a BioLegend 431,301 kit (BioLegend, San Diego, USA). Briefly, a 96-well plate was coated with 100 µL of capture antibody at 4 °C overnight. Then, the plate was washed 4 times using 300 µL of wash buffer. Then, 200 µL of assay diluent was added to each well and incubated at room temperature with shaking for 1 h. The plate was washed 4 times with 300 µL of wash buffer. Then, 100 µL of standard solution and samples were added for incubation at room temperature with shaking for 2 h. The plate was washed 4 times with 300 µL of wash buffer. Then, 100 µL of mouse-IL6 detection antibody solution was added for incubation at room temperature with shaking for 1 h. The plate was washed 4 times using 300 µL of wash buffer. One hundred microliters of avidin-HRP solution were added to each well and incubated at room temperature with shaking for 30 min. The plate was washed 5 times with 300 µL of wash buffer. Then, 100 µL of substrate solution was added and incubated at room temperature with shaking for 15 min. Subsequently, 100 µL of stop solution was added and the absorbance of each well was measured at 450 nm with a microplate reader.

### 3.6. Preparation and Characterization of Hair Scalp Serum Containing PSO-Loaded Niosomes

The optimized formulation of hair scalp serum containing PSO-loaded niosomes is shown in Table 5. Hair scalp serum containing PSO-loaded niosomes was prepared by dispersing acrylates/C10-30 alkyl acrylate crosspolymer in deionized water. Next, glycerin, propylene glycol, and disodium EDTA were added to the above mixture. The preservative (Phenostat^TM^) was added next. Afterwards, tocopheryl acetate, PEG-40 hydrogenated castor oil, and fragrance were added and mixed into the above mixture to form a homogeneous preparation. The active, PSO-loaded niosomes, was then mixed into the formulation at a concentration of 5% w/w. The concentration of PSO-loaded niosomes in the hair scalp serum was higher than the effective concentration determined from the 5α-reductase inhibition and IL-6 inhibition studies. The pH of the formulation was adjusted to 5.0–6.0 by adding 10% *w*/*v* triethanolamine solution. The hair scalp serum containing PSO-loaded niosomes was then characterized in terms of appearance by organoleptic test, pH measured by a FiveEasy Plus FP20 (Mettler Toledo, Greifensee, Switzerland), and viscosity determined by a Haake Roto Visco1 (Thermo Scientific, Karlsruhe, Germany) using rotor type C60/1 TiL with a shear rate of 1000 s^−1^ at 30 °C. The formulation was stored at 40 °C for 3 months to observe its stability. 

### 3.7. Skin Irritation of Hair Scalp Serum Containing PSO-Loaded Niosomes Using Reconstructed Human Skin Model

Skin irritation of hair scalp serum containing PSO-loaded niosomes was studied using a reconstructed human skin model. The reconstructed human epidermis (Episkin^TM^ kit small model) was purchased from Episkin (Lyon, France) and used as a human skin equivalent model. The in vitro skin irritation test was performed according to OECD Test Guideline 439 [40]. This technique had been validated and used to assess the skin irritation potential of chemicals. To simulate the real application of cosmetics, the test sample was applied to the reconstructed human epidermis for 24 h. 

Each transwell of the reconstructed human epidermis (surface area of 0.38 cm^2^) was removed from the nutrient gel and transferred to a sterile 12-well plate containing 2 mL of maintenance medium provided by the manufacturer under aseptic conditions. Then, preincubation was carried out at 37 °C under 5% CO_2_ for 24 h. Afterwards, the test sample (approximately 10 μL) was applied on the apical side of the reconstructed human epidermis for incubation at 37 °C under 5% CO_2_ for 24 h. After a predetermined period, the test sample was washed twice with 15 mL of PBS and transferred to a new 12-well plate filled with 2 mL of maintenance medium. The skin sample was kept in an incubator (37 °C under 5% CO_2_) for a period of 42 ± 1 h. Phosphate-buffered saline (PBS) and 5% sodium dodecyl sulfate were used as negative and positive controls, respectively.

To evaluate the cell viability, each transwell of the reconstructed human epidermis was transferred to a new 12-well plate filled with 2 mL of assay medium containing MTT solution at the concentration of 0.3 mg/mL and further incubated for 3 h (37 °C under 5% CO_2_). After that, the skin was removed from the transwell and formazan crystals were extracted from the skin with acidic isopropanol. The optical density of the formazan product was then measured at 570 nm. The % tissue viability was calculated according to the Equation (1). A percentage of tissue viability of more than 50% indicates that the sample is nontoxic to the cells and does not cause irritation.

### 3.8. Anti-Hair Loss Activity of the Hair Scalp Serum-Containing PSO-Loaded Niosomes

The in vivo anti-hair loss efficacy of the hair scalp serum containing PSO-loaded niosomes was evaluated in panelists. The test protocol was approved by the Faculty of Dentistry/Faculty of Pharmacy, Mahidol University, Institutional Review Board (MU-DT/PY-IRB 2019/016.2603).

#### 3.8.1. Hair Loss Evaluation by 60-Second Hair Count Test

The 60-second hair count experiment was adapted from the study by Wasko et al. [46]. The test was employed to evaluate the anti-hair loss efficacy of the hair scalp serum containing PSO-loaded niosomes. This technique is noninvasive and shows good panelist compliance. The 60-second hair count test was conducted on 42 panelists (20 men and 22 women). All panelists were assigned to use standard shampoo (no active ingredient) once a day and the hair scalp serum containing PSO-loaded niosomes twice daily (morning and evening) for 8 weeks. The 60-second hair count was evaluated by combing the hairs from the back top of the scalp and moving to the front of the scalp for 60 s using the same comb. The number of fallen hairs from each individual panelist at 4 and 8 weeks after using a test product was counted and compared with the number of fallen hairs before treatment.

#### 3.8.2. Hair Loss Evaluation by Phototrichogram Using TrichoScan^®^

Phototrichogram is a noninvasive in vivo technique that allows to determine the physiology of the hair such as hair length, thickness, density, and growth rate. TrichoScan^®^ is comprised of a standard microscope with automatic digital image analysis to evaluate the human hairs. This software can quantify the number of hairs and the ratio of those in anagen and telogen phase (A/T) in a single operation. In this study, 8 panelists (4 men and 4 women) were included to use the standard shampoo (no active ingredient) once a day and the hair scalp serum containing PSO-loaded niosomes twice daily for 8 weeks. The TrichoScan^®^ assay was carried out with the panelists before and after treatment. Initially, the hairs at the selected 1.8 cm^2^ site were trimmed down to a length of 0.5 mm with a special portable trimmer. After 2 days, photographs were taken at the selected site with a digital camera at 20-fold magnification. Then, the obtained images were analyzed by TrichoScan^®^ software, and subsequently the A/T ratio was calculated. The difference between anagen and telogen hairs is on the basis that telogen hairs do not grow. The A/T ratio of each individual panelist after using the scalp hair serum containing PSO-loaded niosomes was compared to that before use at the same scalp area.

### 3.9. Statistical Analysis

The reported data are presented as the mean value ± standard deviation (S.D.). The significance of differences was determined using one-way repeated ANOVA at the probability level of 0.05.

## 4. Conclusions

The present study showed that PSO-loaded niosomes were an effective delivery for the treatment of hair loss. Based on confocal microscopy of pig ear skin, the nanosized PSO-loaded niosomes could penetrate deeper into the skin and accumulate in the hair follicles compared to the oil solution of PSO. The in vitro studies found that the PSO-loaded niosomes inhibited the mRNA expression of genes involved in the synthesis of 5α-reductase and showed anti-inflammatory activity, which was of interest for in vivo hair loss treatment. Hair scalp serum containing PSO-loaded niosomes was prepared and tested for its in vivo efficacy to reduce hair loss. The 60-s hair count study showed a reduction in the percentage of fallen hairs compared to the initial conditions. Additionally, the TrichoScan^®^ assay presented an increased A/T ratio after using the serum for 8 weeks. These in vivo studies illustrated the promising efficacy of hair scalp serum containing PSO-loaded niosomes on the reduction of hair loss and might be further developed as an alternative hair loss treatment.

## Figures and Tables

**Table 1:** Mean and standard deviation of interleukin-6 level and inhibition compared with the positive control evaluated in RAW 264.7 cells.

Sample	IL-6 Detection	
	IL-6 Level (pg/mL)	IL-6 Inhibition Activity over Positive Control (%)
Negative control	2.82 ± 0.27	-
Positive control	627.94 ± 24.35	0.00
PSO-niosomes (100 μg/mL)	36.50 ± 5.38	94.19 ± 0.86
PSO solution (100 μg/mL)	2.80 ± 0.11	99.55 ± 0.02
Diclofenac sodium (1000 µM)	72.46 ± 13.46	88.46 ± 2.14

Data are presented as mean ± standard deviation. Inhibitory activity over positive control was measured based on the comparison between treatment and positive control.

**Table 2:** The average number and percent reduction of fallen hairs compared to the initial conditions from the 60-second hair count study on 42 panelists after using hair scalp serum containing PSO-loaded niosomes for 4 and 8 weeks.

Time	Average Number of Fallen Hairs	% Reduction of Fallen Hairs	*p*-Value
Initial	9.60 ± 5.08	-	-
4 weeks	7.33 ± 4.64	23.57 ± 1.29	0.024 *
8 weeks	5.33 ± 3.11	44.42 ± 1.22	0.000 *

* The 60-second hair count was significantly different from the initial analysis using one-way repeated ANOVA at *p* < 0.05.

**Table 3:** Mean and standard deviation of interleukin-6 level and inhibition compared with the positive control evaluated in RAW 264.7 cells.

Time	% Anagen	% Telogen	A/T Ratio	*p*-Value
Initial	74.53 ± 9.38	25.81 ± 9.85	3.48 ± 2.06	
4 weeks	78.45 ± 5.74	21.55 ± 5.74	3.92 ± 1.22	1.000
8 weeks	80.98 ± 6.04	19.03 ± 6.04	4.72 ± 1.76	0.036 *

* A/T ratio was significantly different from the initial, analysis using one-way repeated ANOVA at *p* < 0.05.

**Table 4:** The designed gene-specific primers for RT–qPCR.

Gene Specific Primers		Sequences
Human GAPDH	sense antisense	5′-CAAATTCCATGGCACCGTCA-3′ 5′-ATCGCCCCACTTGATTTTGG-3′
Human SRD5A1	sense antisense	5′-TGGGGATAGAGGAGGAAGCT-3′ 5′-GTATGAACCACCACCAGCAC-3′
Human SRD5A2	sense antisense	5′-CAGAGCCCACATTTCCACAC-3′ 5′-GCCCCTTCCTTAGAGAGTCC-3′

GAPDH is glyceraldehyde 3-phosphate dehydrogenase; SRD5A1 is steroid 5α-reductase 1; SRD5A2 is steroid 5α-reductase 2.

**Table 5:** The composition of the optimized hair scalp serum containing PSO-loaded niosomes.

Ingredient	Master Formula (% w/w)
Deionized water	88.05
Acrylates/C10-30 alkyl acrylate crosspolymer	0.30
Glycerin	1.00
Propylene glycol	1.00
Disodium EDTA	0.20
Phenostat	1.00
Tocopheryl acetate	0.20
PEG-40 hydrogenated castor oil	1.00
Fragrance	0.05
PSO-loaded niosomes	5.00
10% *w*/*v* triethanolamine solution	q.s.

Note: q.s., stands for quantity sufficient.

## Data Availability

Data is contained within the article.
